# Cultural differences in positive psychotic experiences assessed with the Community Assessment of Psychic Experiences-42 (CAPE-42): a comparison of student populations in the Netherlands, Nigeria and Norway

**DOI:** 10.1186/s12888-019-2210-8

**Published:** 2019-08-06

**Authors:** Margriet Vermeiden, Mayke Janssens, Viviane Thewissen, Esther Akinsola, Sanne Peeters, Jennifer Reijnders, Nele Jacobs, Jim van Os, Johan Lataster

**Affiliations:** 1 Faculty of Psychology and Educational Sciences, Open University, Valkenburgerweg 177, Heerlen, 6419 AT The Netherlands; 2 Department of Psychiatry and Psychology, School for Mental Health and Neuroscience, Maastricht University Medical Centre, Maastricht, The Netherlands; 3 Department of Psychology, Faculty of Social Sciences, University of Lagos, Akoka, Lagos Nigeria; 4 Department of Psychiatry, Brain Centre Rudolf Magnus, University Medical Centre Utrecht, Utrecht, The Netherlands; 5 Department of Psychosis Studies, Institute of Psychiatry, psychology and Neuroscience, King’s College, London, UK

**Keywords:** Community Assessment of Psychic Experiences, Psychosis, Schizophrenia, Mental illness, Stress, Epidemiology, General population, Cross-national, Cross-cultural

## Abstract

**Background:**

Previous studies have suggested that culture impacts the experience of psychosis. The current study set out to extend these findings by examining cultural variation in subclinical positive psychotic experiences in students from The Netherlands, Nigeria, and Norway. Positive psychotic experiences were hypothesized to (i) be more frequently endorsed by, and (ii) cause less distress in Nigerian vs. Dutch and Norwegian students.

**Methods:**

Psychology students, aged 18 to 30 years, from universities in the Netherlands (*n* = 245), Nigeria (*n* = 478), and Norway (*n* = 162) were assessed cross-sectionally with regard to the frequency of subclinical positive psychotic experiences and related distress, using the Community Assessment of Psychic Experiences (CAPE-42). Multi-group confirmatory factor analysis and multivariate analysis of covariance were performed to assess measurement invariance of the positive symptom dimension (CAPE-*Pos*) and compare mean frequency and associated distress of positive psychotic experiences across study samples.

**Results:**

Only CAPE-*Pos* items pertaining to the dimensions ‘strange experiences’ and ‘paranoia’ met assumptions for (partial) measurement invariance. Frequencies of these experiences were higher in the Nigerian sample, compared to both the Dutch and Norwegian samples, which were similar. In addition, levels of experience-related distress were similar or higher in the Nigerian sample compared to respectively the Dutch and Norwegian samples.

**Conclusion:**

Although positive psychotic experiences may be more commonly endorsed in non-Western societies, our findings do not support the notion that they represent a more benign, and hence less distressing aspect of human experience. Rather, the experience of psychotic phenomena may be just as, if not more, distressing in African than in European culture. However, observed differences in CAPE-*Pos* frequency and distress between samples from different cultural settings may partly reflect differences in the measure rather than in the latent trait. Future studies may therefore consider further cross-cultural adaptation of CAPE-42, in addition to explicitly examining cultural acceptance of psychotic phenomena, and environmental and other known risk factors for psychosis, when comparing and interpreting subclinical psychotic phenomena across cultural groups.

## Background

The psychosis phenotype appears to become manifest across a spectrum of severity, extending from subtle psychotic experiences to a clinically relevant disorder [1, 2]. Although epidemiological studies have emphasized similarities, rather than differences in the prevalence of psychotic disorders across cultures [3–8], there are cultural variations in the manifestation of psychotic experiences [9, 10] with as much as 15–30% of psychosis expression being suggested to be culture-dependent [11]. The sociocultural context in which psychotic symptoms manifest themselves, and the associated degree of preoccupation and distress are important factors determining clinical relevance, but have not been investigated much [12]. Given that subclinical psychotic experiences may predict transition to a clinically relevant psychotic disorder, knowledge of cultural diversity in manifestations of psychotic experiences is important for the proper contextual assessment and treatment of psychotic disorders [13, 14]. Thus, an increasing number of studies explore cultural mechanisms that play a role in the expression of psychopathology [15].

Cultural representations, such as values, beliefs, and attitudes, establish a general understanding of the concept of reality, and influence how members of a particular society respond to psychotic phenomena [16]. The common societal conception of values and beliefs appears to be particularly important for the theme of delusions and hallucinations [17]. Delusions of grandeur, for example, hardly exist in societies where striving for a certain social status is frowned upon [11], and visual and tactile hallucinations are reported more frequently by members of social groups that take unexplainable sensory experiences as evidence of the supernatural or divine [16, 18]. These examples illustrate that the likelihood and quality of psychotic phenomena is partly dependent on an individual’s cultural environment [13, 19, 20]. Moreover, the extent to which hallucinations and delusions are interpreted as appropriate or benign, and socially accepted, may have implications for the distress resulting from these experiences within a certain society (i.e., distress is likely lower when experiences are considered normative or appropriate; [16, 20–24]).

To better comprehend cross-cultural differences, several studies suggest that positive psychotic experiences are not to be interpreted as homogeneous dimensions, but are better represented as a number of symptom clusters that may vary in prevalence across cultures [25–31]. Indeed, a number of cross-national survey studies have reported considerable variation in the prevalence of particular psychotic experiences across groups of individuals from different parts of the world, in the absence of any relevant differences in the overall prevalence of psychotic disorder [32–36]. Similar findings have been reported with regard to the prevalence of positive symptom clusters across different ethnic groups within certain countries: disparity in symptom prevalence between ethnic groups exceeds disparity in prevalence of psychotic disorder [37–39]. This seems to be especially true for perceptual anomalies and paranoia [40–42]. Moreover, discrepancies between positive symptom prevalence and prevalence of a diagnosis of psychosis have been shown to be more pronounced in some ethnic groups than in others [10, 37, 39, 43], pointing towards a complex interplay of cultural and social factors (e.g. ethnic minority status) in the manifestation of psychosis [44–47].

The majority of studies assessing cross-cultural differences in prevalence of psychotic experiences have employed trained clinical interviewers or used highly structured interviews, developed in accordance with definitions and criteria of ICD-10 and/or DSM-(I)V classification systems for psychotic disorder [48]. The Community Assessment of Psychic Experiences (CAPE-42) [49]–a 42-item self-report questionnaire–on the other hand, was developed specifically to measure psychosis proneness in the general population based on a severity spectrum view of the psychosis phenotype. The CAPE-42 has proven to be a stable, reliable and valid instrument [49]. A recent meta-analysis of studies employing CAPE-42 [25] supported three dimensions of positive psychotic experiences in the general population, namely ‘strange experiences’, ‘delusional ideations’ (including paranoia, grandiosity and paranormal beliefs), and ‘perceptual anomalies’ (i.e., hallucinations). Although this meta-analysis [25] included samples from different parts of the world, the focus was mainly on identifying general underlying symptom structures, rather than comparing symptom prevalence across cultural groups or study samples. Moreover, the reviewed samples were from the so-called Western World, and to date CAPE-42 psychotic experiences have been rarely investigated in African study samples [22, 42].

The current study, therefore, aimed at furthering insight into the cultural variation in positive psychotic experiences as measured by CAPE-42, by comparing prevalence and perceived distress of these experiences across student populations from universities in The Netherlands, Norway (i.e., Western and Northern Europe) and Nigeria (Yoruba homeland). The distinction between reality and fantasy has been described as being more rigid in Euro-American culture in comparison to non-Western societies, and reports of psychotic-like phenomena (e.g. paranormal beliefs, feeling the presence of and/or being controlled by a deceased family member or under the influence of a supernatural force) are therefore more likely to be labelled as pathological in Western settings [50]. A greater degree of cultural acceptance towards such phenomena outside of the Western setting may lead to more frequent endorsement of psychotic experiences [16], as supported previously by a number of cross-national reports on the prevalence of hallucinations and delusions [13, 51–55]. Recently, two large studies [22, 42] demonstrated cultural variation in epidemiological expression of psychotic experiences across respectively 12 and 13 countries, with highest prevalence rates in African samples. In line with these findings, we hypothesized higher prevalence of subclinical CAPE-42 psychotic experiences and lower prevalence of related distress in the Nigerian sample, compared to the Dutch and Norwegian samples. With regard to differences between the latter two samples, in Northern Europe living in the high-latitude band has been associated with higher incidence rates of psychotic disorders (the ‘latitude effect’ [56]). However, this effect possibly represents an artefact of methodological inconsistencies between studies (especially, use of register-based vs. first-contact data [57]), as there is little evidence of actual country variation in prevalence and distress of mental disorders across Europe when these inconsistencies are accounted for (see critical review by [58]). Although findings are thus inconclusive, we did not expect (pronounced) differences in prevalence and distress rates of subclinical positive psychotic experiences between Dutch and Norwegian samples, based on the assumption that participants from these countries would hold similar beliefs regarding the distinction between reality and fantasy, despite other cultural differences. In sum, we thus expected reports of hallucinations, strange experiences, and delusional ideations to be more prevalent in the Nigerian sample and, as theorized previously [16, 22, 50], the average distress related to these experiences to be relatively lower in the Nigerian sample, compared to both the Dutch and Norwegian samples. The lower prevalence of distress was based on the idea that these experiences are more likely labelled as appropriate or benign within African cultures, and hence met with more constructive attitudes [16, 20–24, 42, 50, 59, 60].

## Methods

### Sample

The non-probability sample for this study, to which we had access, consisted of 885 psychology students from the Open University of the Netherlands (*n* = 245), the University of Lagos, Nigeria (*n* = 478), and the Universities of Oslo, Trondheim and Bergen, Norway (*n* = 162). Inclusion criteria were: (i) aged 18–30 years old, (ii) sufficient command of the Dutch, English or Norwegian language, respectively, to understand instructions and give informed consent, and (iii) no current or lifetime history of psychotic disorder. The total sample consisted of 307 males and 576 females, ranging in age from 18 to 30 years (*Mean* = 23.3, *SD* = 3.3). Further demographic characteristics of the sample are displayed in Table 1.

**Table 1:** Demographic characteristics and unadjusted descriptives of CAPE-*Pos* by sample, and tests for between-group differences

	Netherlands (*n* = 245)	Nigeria (*n* = 478)	Norway (*n* = 162)	*F /* χ^2a^ (R^2^)	*p*
Age, *M* (*SD*)	25.7 (3.0) range 18–30	22.0 (2.9) range 18–30	23.3 (2.8) range 18–30	*F*(2,882) = 135.36 (R^2^ = 0.235)	< 0.001
Gender, *n* (%)				χ^2^(2, *n* = 883) = 74.55^b^	< 0.001
Male	50 (20.4%)	227 (47.5%)	30 (18.5%)		
Female	193 (78.8%)	251 (52.5%)	132 (81.5%)		
Missing	2 (0.8%)	–	–		
Marital status, *n* (%)				χ^2^(2, *n* = 883) = 54.56^b^	< 0.001
Not Married	195 (79.6%)	461 (96.4%)	151 (93.2%)		
Married	48 (19.6%)	17 (3.6%)	11 (6.8%)		
Missing	2 (0.8%)	–	–		
Education^c^, *n* (%)				χ^2^(2, *n* = 878) = 219.60	< 0.001
Secondary	26 (10.6%)	318 (67.5%)	101 (62.3%)		
Tertiary	219 (89.4%)	153 (32.5%)	61 (37.7%)		
Ethnic group, *n* (%)^d^	–		–	–	–
Yoruba	–	327 (68.4%)	–		
Igbo	–	80 (16.7%)	–		
Hausa	–	8 (1.7%)	–		
Other / missing	–	63 (13.2%)	–		
CAPE-*Pos total*					
Frequency, *M (SD)*	1.37 (0.21)	1.98 (0.40)	1.31 (0.19)	*F*(2,882) = 431.29	< 0.001
(min – max)	(1.00–2.20)	(1.13–3.16)	(1.05–2.10)	(R^2^ = 0.494)	
Distress^e^, *M (SD)*	1.53 (0.42)	1.78 (0.53)	1.36 (0.42)	*F*(2,874) = 50.35	< 0.001
(min – max)	(1.00–3.20)	(1.00–3.21)	(1.00–3.00)	(R^2^ = 0.103)	
Distress^f^, *M (SD)*	1.14 (0.14)	1.54 (0.47)	1.12 (0.14)	*F*(2,881) = 141.42	< 0.001
(min – max)	(1.00–1.70)	(1.00–3.50)	(1.00–1.65)	(R^2^ = 0.243)	

^a^Chi-square and ANOVA tests were performed to test whether the distribution of demographic and CAPE-*Pos* scores was comparable across the three study samples, ^b^Based on 2 × 3 table (not including missing category), ^c^Highest educational level completed, ^d^Only assessed in Nigerian sample, ^e^Distress scores when frequency of experience was rated as at least ‘sometimes’, ^f^Distress including scores when frequency of experience was rated as ‘never’ (following [25, 61, 62])

### Study design and procedure

The study employed a cross-sectional survey design, asking participants to fill out a paper (Nigeria) or online self-report questionnaire (Netherlands and Norway). A lecturer of the University of Lagos directly approached Nigerian participants in 2011, handing out surveys during classes and giving participants the opportunity to directly give verbal consent and fill out the questions, which resulted in a 100% return rate. Dutch participants were recruited in 2009, and received course credits for their participation. The survey return rate was 100%. Norwegian participants were recruited through local university email services, electronic advertisements and Facebook in 2010 and, as a consequence, the total sampling frame and survey response rate are unknown. A total number of 246 Dutch, and 162 Norwegian psychology students accessed the online survey webpage, of which respectively 243 (98.8%), and 158 (97.5%) provided a complete survey response (i.e., all demographic and CAPE-42 items answered). A total number of 501 Nigerian psychology students handed in a paper survey document, of which 316 (63.1%) provided a complete response. We followed previous work on CAPE psychotic-like experiences [30], and excluded all participants with ≥ 25% CAPE-42 data missing, thereby eliminating data rows from 24 participants (< 3% of total recruited sample; one Dutch, and 23 Nigerian participants). The final dataset for analysis therefore contained data from *N* = 885 individuals, of which 771 (87.1%) provided complete demographic and CAPE-42 data, 109 (12.3%) had no more than five missing values on the set of CAPE frequency variables for analysis, and five individuals (0.6%) had more than five but less than 25% missing data points.

### Measures

The Community Assessment of Psychic Experiences is a 42-item self-report instrument that measures the lifetime frequencies and distress of positive, negative and depressive subclinical psychotic experiences [63]. CAPE-42 has proven to be an appropriate tool to assess these experiences in clinical and subclinical populations [25], and has been cross-validated with the Structured Interview for Schizotypy (SIS-R) and the Brief Psychiatric Rating Scale (BPRS), demonstrating good stability, reliability and (discriminant) validity [49]. The current study used validated Dutch and English versions of the instrument [64, 65], and a validated Norwegian translation [66]. The Norwegian [66] and English validation [65] of CAPE-42 suggested sufficient internal consistency of positive, negative and depressive experiences, with Cronbach’s alpha values of 0.87, 0.87, 0.82 (Norway), and 0.83, 0.81, 0.84 (Canada), respectively. The Dutch version of CAPE-42 showed acceptable 3-factor model fit (positive, negative, depressive experiences) with RMSEA = 0.05 and TLI = 0.94, though not fully supported by CFI = 0.81 [64].

The positive symptom dimension (hereafter: CAPE-*Pos*) that is more closely examined in the current study consists of scores on 20 items, asking participants about subclinical positive psychotic experiences (e.g. “Do you ever hear voices when you are alone?”, “Do you ever feel as if you are under the control of some power other than yourself?”, “Do you ever feel as if there is a conspiracy against you?”). The frequency of each experience is rated on a four-point scale, ranging from 1 (*never*), 2 (*sometimes*), 3 (*often*), to 4 (*nearly always*), and a four-point scale from 1 (*not distressed*), 2 (*a bit distressed*), 3 (*quite distressed*) to 4 (*very distressed*) measures the degree of associated distress for each experience that is labelled as present (frequency of at least *sometimes*). The CAPE-*Pos* scale was initially conceptualized as unidimensional [67], but later studies have suggested either a three-dimensional structure that distinguishes ‘hallucinations’ (4 items), ‘strange experiences’ (7 items), and ‘delusional ideations’ (9 items; [25]), or a five-dimensional structure, in which the delusional ideation dimension is further decomposed into the subdimensions ‘paranoia’ (5 items), ‘grandiosity’ (2 items), and ‘magical thinking’ (2 items; [26]).

### Analysis

Data were analysed with R version 3.5.3 [68], using the *psych* (version 1.8.12 [69]) and *lavaan* packages (version 0.6–3 [70]), and SPSS Statistics version 25.0 [71]. Data were inspected for anomalies, but outliers were not removed, as extreme scores were argued to indicate actual severe psychotic experiences.

Demographic variability in frequency and distress of CAPE-42 positive psychotic experiences was preliminary explored using multiple regression analyses with weighted mean frequency and distress scores for all CAPE-*Pos* items as dependent variables and age, gender (1 = male; 2 = female), marital status (1 = not married; 2 = married), and educational level (1 = secondary education; 2 = tertiary education) as independent variables.

Tests for configural, metric, and scalar invariance were performed to investigate the degree of measurement invariance for CAPE-*Pos* frequency ratings across groups, with (partial) invariance being considered as prerequisite for validly interpreting mean comparisons between samples as reflecting differences in the latent construct or trait rather than the measure [72–74]. In line with previous work [64], measurement invariance testing for the distress ratings was omitted, as these values were selectively available only for those experiences labelled as present (frequency of at least *sometimes*).

Configural invariance was assessed by performing maximum likelihood confirmatory factor analysis (CFA) to investigate the fit of one-, three-, and five-dimensional models of CAPE-*Pos* [25, 26, 67] in the whole sample, and in the Dutch, Nigerian, and Norwegian samples separately. Comparative fit index (CFI), root mean square error of approximation (RMSEA), and the standardized root mean square residual (SRMR) were computed for absolute goodness of fit exploration (minimally acceptable fit criteria: CFI ≥ 0.90, [75]; RMSEA ≤ 0.08, [76]; SRMR ≤ 0.08, [77]), and Akaike information criterion (AIC) and sample size adjusted Bayesian information criterion (BIC) were used to compare relative goodness of fit (smaller values indicate better fit). Internal consistency for the initial CFA-supported CAPE-*Pos* dimensions was assessed in each sample using McDonald’s ω [78], and inter-item correlations (IIC) for dimensions consisting of two items.

Configural, metric and scalar invariance of CAPE-*Pos* scores across Dutch, Nigerian, and Norwegian samples were further assessed by performing maximum likelihood multi-group confirmatory factor analysis (MGCFA [79]), in which several invariance models were compared sequentially, each model adding more restrictions and being tested against the less constrained model [73, 74, 80]. Specification of the baseline configural model was based on CFA results. Metric invariance was tested by constraining factor loadings, and comparing fit to the baseline model, in which loadings were allowed to be freely estimated across groups. The scalar invariance model additionally constrained item intercepts, and was tested against the metric invariance model. χ^2^ difference tests and ΔCFI were used to examine differences between the nested models, with ΔCFI < 0.01 implying that the invariance assumption holds [81]. When full invariance was not supported, modification indices were explored to identify non-invariant items, and constraints for these items were lifted, thus allowing partial invariance [72, 82].

When at least partial scalar invariance was supported, multivariate analyses of covariance (MANCOVAs) were performed to test for mean differences in the frequency and associated distress of CAPE-*Pos* experiences between Dutch, Nigerian and Norwegian participants. In order to do so, for those CAPE-*Pos* dimensions meeting criteria for (partial) measurement invariance, raw scores on CAPE-*Pos* frequency and distress items were transformed into weighted means (i.e. sum of item scores divided by the number of items with valid responses). Weighted means for experience related distress were only calculated when at least one positive psychotic experience within the respective CAPE-*Pos* dimension was labelled as present (frequency of at least *sometimes*). Covariates in the MANCOVA models were age, gender, marital status, and educational level. Whenever the MANCOVA tests yielded significant results, pairwise comparisons of estimated marginal means were conducted to explore group differences with Bonferroni corrected statistics, and Hedges’ g values were calculated by dividing mean differences between groups by the pooled and weighted standard deviation [83].

All findings were interpreted against a significance threshold of *p* <  0.05, and the respective n for each analysis is presented along with model estimates. (MG)CFAs were performed with full information maximum likelihood (FIML) estimation to account for missing data points [70, 84], but were additionally repeated using listwise deletion (results presented in Appendix) to ensure that findings were not systematically biased by missing data processing.

## Results

### Sample characteristics and demographic variability in CAPE-Pos frequency and distress

Table 1 presents a demographic description of the Dutch, Nigerian, and Norwegian samples, together with mean frequency and distress scores for CAPE-*Pos*. Dutch participants were oldest, most often married, and had generally completed tertiary educational level. By contrast, Nigerian participants were youngest, generally not married, and had typically not (yet) completed the tertiary educational level. Women were overrepresented in the Dutch and Norwegian samples, whereas in the Nigerian sample male and female participants were represented equally. Among the Nigerian participants, 68.4, 16.7, and 1.7% considered themselves as belonging to the Yoruba, Igbo, or Hausa ethnic group, respectively.

In the total sample, preliminary multiple regression analysis revealed that CAPE-*Pos* frequency scores were significantly associated with age, gender, and educational level (F_(4,871)_ = 49.37, *p* <  0.001; R^2^ = 0.185): higher CAPE-*Pos* frequency was linked to younger (vs. older; B(SE) = − 0.04(0.005), p <  0.001), male (vs. female; B(SE) = − 0.201(0.030), p <  0.001) and lower (vs. higher; B(SE) = − 0.076(0.031), *p* = 0.014) educated participants. CAPE-*Pos* distress scores were significantly associated with age and gender (F_(4,870)_ = 15.98, p <  0.001; R^2^ = 0.069): higher CAPE-*Pos* distress was linked to younger (vs. older; B(SE) = − 0.025(0.005), p <  0.001), and male (vs. female; B(SE) = − 0.106(0.029), p <  0.001) participants.

### Confirmatory factor analysis and internal consistency estimates

Fit indices for one-, three-, and five-dimensional models of CAPE-*Pos* are presented in Table 2. In the total sample, only the five-dimensional model fulfilled all three fit criteria for CFI, SRMR and RMSEA. In the Dutch and Nigerian samples, the five-dimensional model best fit the data: AIC and BIC values were lower compared to those of the three- and one-dimensional models, SRMR and RMSEA reached threshold values, and CFIs were 0.845 (Dutch sample) and 0.902 (Nigerian sample). In the Norwegian sample, none of the tested models reached threshold values for CFI, SRMR and RMSEA, indicating poor or suboptimal fit of previously suggested CAPE-*Pos* factor structures in the Norwegian sample. Thus, CFA largely supported five-factor structures of CAPE-*Pos* in the Dutch and Nigerian samples, but not in the Norwegian sample. Repeating CFAs using listwise deletion instead of FIML returned slightly lower CFI-values, but yielded similar results overall (see Table 7 in Appendix).

**Table 2:** Confirmatory Factor Analysis for the total sample, and for the three study samples separately

CAPE-*Pos* frequency	χ^2a,b^	df^c^	*p*	Absolute goodness of fit			Comparative fit		
				RMSEA^d^ (90% CI)	CFI^d^	SRMR	AIC^b^	BIC^b,e^	Factor loadings
Total sample (*n* = 885)^f^									
1-factor model^g^	789	152	< 0.001	0.084 (0.078; 0.090)	0.821	0.059*	34089	34181	0.315–0.697
3-factor model^h^	543	149	< 0.001	0.066 (0.060; 0.072)*	0.891	0.049*	33715	33812	0.328–0.810
5-factor model^i^	356	142	< 0.001	0.050 (0.043; 0.056)*	0.941*	0.040*	33457	33565	0.350–0.902
Dutch sample (*n* = 245)^f^									
1-factor model^g^	344	152	< 0.001	0.082 (0.071; 0.094)	0.573	0.085	4035	4048	0.029–0.541
3-factor model^h^	317	149	< 0.001	0.074 (0.063; 0.086)*	0.656	0.083	3973	3987	0.278–0.679
5-factor model^i^	218	142	< 0.001	0.051 (0.037; 0.064)*	0.845	0.068*	3868	3884	0.299–0.988
Nigerian sample (*n* = 478)^f^									
1-factor model^g^	473	152	< 0.001	0.073 (0.066; 0.081)*	0.770	0.064*	20477	20534	0.039–0.615
3-factor model^h^	363	149	< 0.001	0.060 (0.052; 0.068)*	0.849	0.058*	20344	20404	0.062–0.756
5-factor model^i^	281	142	< 0.001	0.049 (0.041; 0.058)*	0.902*	0.048*	20260	20327	0.242–0.761
Norwegian sample (*n* = 162)^f^									
1-factor model^g^	362	152	< 0.001	0.101 (0.088; 0.114)	0.452	0.099	3954	3950	0.182–0.534
3-factor model^h^	319	149	< 0.001	0.087 (0.074; 0.101)	0.596	0.090	3876	3871	0.181–0.945
5-factor model^i^	303	142	< 0.001	0.086 (0.073; 0.100)	0.624	0.088	3866	3862	0.212–0.971

^a^Yuan-Bentler scaled test-statistic, ^b^Rounded to nearest integer, ^c^Item no. 41 dropped, due to (near-)zero variance in Dutch and Norwegian samples (i.e. (virtually) all scores = 1), ^d^Robust RMSEA and CFI from the scaled test-statistic, ^e^Sample size adjusted Bayesian BIC, ^f^Missing datapoints were processed using full information maximum likelihood estimation, ^g^CAPE-*Pos* one-factor structure as originally reported by [67], ^h^CAPE-*Pos* three-factor structure as reported by [25], ^i^CAPE-*Pos* five-factor structure as reported by [26], *Meeting minimally acceptable fit criteria: CFI ≥ 0.90 [75], RMSEA ≤ 0.08 [76], SRMR ≤ 0.08 [77]

Internal consistency estimates for the five CAPE-*Pos* dimensions–‘hallucinations’, ‘strange experiences’, ‘paranoia’, ‘grandiosity’, and ‘magical thinking’–that were largely supported by CFA in the Dutch and Nigerian samples were, respectively, ω = 0.53, ω = 0.74, ω = 0.30, IIC = 0.58, IIC = 0.40 in the Dutch sample, ω = 0.75, ω = 0.82, ω = 0.63, IIC = 0.36, IIC = 0.09 in the Nigerian sample, and ω = 0.69, ω = 0.68, ω = 0.59, IIC = 0.38, IIC = 0.22 in the Norwegian sample.

### Metric and scalar invariance

Fit statistics for different invariance models of CAPE-*Pos* across study samples are presented in Table 3. In line with CFA results, the five-dimensional configural invariance (baseline) model did not fit the data well, and assumptions of metric and scalar invariance were violated. Modification indices were explored to identify non-invariant items, and it was concluded that only those items assigned to the dimensions ‘strange experiences’ and ‘paranoia’ were (largely) invariant across samples. The configural (baseline) model was respecified accordingly, yielding improved fit, and full metric and partial scalar invariance were established across study samples for the redefined, reduced model (see Table 3 for details). Repeating MGCFA using listwise deletion instead of FIML returned slightly lower fit values for all models, but provided similar results regarding metric and scalar invariance between samples (see Table 8 in Appendix).

**Table 3:** Fit statistics for different invariance models of CAPE-42 positive psychotic experiences across study samples

CAPE-*Pos* frequency	χ^2^ (Δχ^2^)^a^	df (Δdf)	*p* (Δ*p*)	CFI (ΔCFI)^b^	RMSEA (ΔRMSEA)^b^	SRMR (ΔSRMR)	Comparison	Decision
5-dimensional model^c^; *n* = 245^d^ (Netherlands); *n* = 478^d^ (Nigeria); *n* = 162^d^ (Norway).								
M1 Configural invariance	798	426	< 0.001	0.843	0.058	0.060	–	Reject
M2 Metric invariance	840 (42)	454 (28)	< 0.001 (0.012)	0.825 (0.018)	0.060 (−0.002)	0.071 (−0.011)	M1 vs. M2	Reject
M3 Scalar invariance	1053 (213)	482 (28)	< 0.001 (< 0.001)	0.740 (0.085)	0.071 (− 0.011)	0.087 (− 0.016)	M2 vs. M3	Reject
2-dimensions reduced model (Strange experiences – items 5, 17, 24, 26, 28, 30, 31; Paranoia – items 2, 6, 7, 22); *n* = 245^d^ (Netherlands); *n* = 478^d^ (Nigeria); *n* = 162^d^ (Norway).								
M1 Configural invariance	198	129	< 0.001	0.924	0.048	0.048	–	Accept
M2 Metric invariance	217 (19)	147 (18)	< 0.001 (0.206)	0.915 (0.009)	0.048 (0.00)	0.062 (−0.014)	M1 vs. M2	Accept
M3 Scalar invariance	341 (124)	165 (18)	< 0.001 (< 0.001)	0.787 (0.128)	0.071 (−0.023)	0.086 (−0.024)	M2 vs. M3	Reject
M3a Partial scalar invariance^e^	231 (14)	159 (12)	< 0.001 (0.300)	0.912 (0.003)	0.046 (0.002)	0.065 (−0.003)	M2 vs. M3a	Accept

ΔCFI < 0.01 implies that invariance assumption holds [81], ^a^Yuan-Bentler scaled test-statistic (rounded to nearest integer), ^b^Robust RMSEA and CFI from the scaled test-statistic, ^c^Item no. 41 dropped, due to (near-)zero variance in Dutch and Norwegian samples (i.e. (virtually) all scores = 1), ^d^Missing data points were processed using full information maximum likelihood estimation, ^e^No intercept constraints for items 6,7,17

### Mean differences in CAPE-pos frequency and associated distress between groups

Based on results from MGCFA, tests for mean differences in the frequency and associated distress of CAPE-*Pos* experiences between samples were limited to the CAPE-*Pos* dimensions ‘strange experiences’ and ‘paranoia’. In nearly all MANCOVA models, the assumption of homogeneity of variance was violated (Levene’s tests were significant), and bootstrapping was used to correct the confidence interval for bias. Samples differed significantly with regard to the mean frequency of ‘strange experiences’ and ‘paranoia’ (Wilk’s *Λ* = 0.748, *F*(4, 1736) = 67.74, *p* <  0.001; see Table 5). Post-hoc analyses showed that estimated marginal means for the frequency of ‘strange experiences’ and ‘paranoia’ were significantly higher in the Nigerian sample (all comparisons *p* <  0.001), compared to the Dutch and Norwegian samples, which were similar (see Tables 4 and 6).

**Table 4:** Estimated marginal means for frequency and distress of CAPE-42 strange experiences and paranoia

CAPE-*Pos*	Frequency						Distress^a^					
	Netherlands		Nigeria		Norway		Netherlands		Nigeria		Norway	
	*n*	*M (SE)* ^*b*^	*n*	*M (SE)* ^*b*^	*n*	*M (SE)* ^*b*^	*n*	*M (SE)* ^*b*^	*n*	*M (SE)* ^*b*^	*n*	*M (SE)* ^*b*^
Strange experiences^c^	243	1.146 (0.032)	471	1.738 (0.021)	162	1.188 (0.033)	106	1.326 (0.074)	365	1.842 (0.035)	80	1.299 (0.072)
Paranoia^c^	243	1.654 (0.045)	471	2.004 (0.030)	162	1.806 (0.047)	106	1.999 (0.083)	365	1.836 (0.039)	80	1.588 (0.080)

^a^Only including distress scores when frequency of experience was rated as at least ‘sometimes’, ^b^Adjusted for age, gender, marital status, and educational level, ^c^Partial scale

The multivariate model of CAPE-*Pos* distress associated with ‘strange experiences’ and ‘paranoia’ also revealed significant differences between samples, Wilk’s *Λ* = 0.869, *F*(4, 1086) = 19.81, *p* <  0.001 (See Table 5). Post-hoc comparison of estimated marginal means showed that distress associated with ‘strange experiences’ was significantly higher in the Nigerian sample (all comparisons *p* <  0.001), compared to the Dutch and Norwegian samples, which were similar. Distress associated with ‘paranoia’ was similar in Dutch and Nigerian participants, and significantly higher compared to Norwegian participants (see Tables 4 and 6).

**Table 5:** Between-group analysis of frequency and associated distress of CAPE-42 strange experiences and paranoia

CAPE-*Pos*	*Frequency*				*Distress* ^*a*^			
	*F* ^*b*^	*df*	*p*	*η*_*p*_^*2*^	*F* ^*b*^	*df*	*p*	*η*_*p*_^*2*^
Strange experiences^c^	143.16	(2,869)	< 0.001	0.248	31.85	(2,544)	< 0.001	0.105
Paranoia^c^	18.72	(2,869)	< 0.001	0.041	6.82	(2,544)	0.001	0.024

All models adjusted for age, gender, marital status, and educational level, ^a^Only including distress scores when frequency of experience was rated as at least ‘sometimes’, ^b^Multivariate *F* ratios were generated from Wilk’s statistics, ^c^Partial scale

**Table 6:** Pairwise comparisons for frequency and associated distress of CAPE-42 strange experiences and paranoia between samples

CAPE-*Pos*	Netherlands (reference) vs. Nigeria			Netherlands (reference) vs. Norway			Nigeria (reference) vs. Norway		
	ΔM (SE) (95% CI)^a^	*p* ^b^	Hedges’ g	ΔM (SE) (95% CI)^a^	*p* ^b^	Hedges’ g	ΔM (SE) (95% CI)^a^	*p* ^b^	Hedges’ g
Frequency									
Strange experiences^c^	0.593 (0.042) (0.492; 0.694)	< 0.001	1.26	0.042 (0.047) (−0.070; 0.154)	0.999	0.09	−0.551 (0.040) (−0.645; − 0.455)	< 0.001	1.23
Paranoia^c^	0.351 (0.059) (0.209; 0.492)	< 0.001	0.52	0.152 (0.065) (−0.005; 0.309)	0.061	0.23	−0.198 (0.056) (− 0.333; − 0.064)	0.001	0.31
Distress^d^									
Strange experiences^c^	0.516 (0.089) (0.304; 0.729)	< 0.001	0.75	−0.027 (0.103) (− 0.274; 0.221)	0.999	0.04	−0.543 (0.080) (− 0.736; − 0.350)	< 0.001	0.82
Paranoia^c^	−0.163 (0.099) (− 0.400; 0.074)	0.295	0.21	− 0.411 (0.115) (− 0.687; − 0.135)	0.001	0.51	−0.248 (0.090) (− 0.463; − 0.033)	0.018	0.34

^a^Based on estimated marginal means, and adjusted for age, gender, marital status, and educational level, ^b^Bonferroni adjustment for multiple comparisons, ^c^Partial scale, ^d^Only including distress scores when frequency of experience was rated as at least ‘sometimes’

## Discussion

This study examined cultural variation in manifestations of positive psychotic experiences as measured by CAPE-42 in student populations from universities in the Netherlands, Nigeria (Yoruba homeland) and Norway. Findings showed that only those items of CAPE-*Pos* pertaining to the dimensions ‘strange experiences’ and ‘paranoia’ (largely) met assumptions for measurement invariance across samples, whereas those that were labelled as belonging to dimensions of ‘hallucinations’, ‘grandiosity’, and ‘magical thinking’ in previous studies did not. For those positive psychotic experiences that could be validly compared between samples (i.e., ‘strange experiences’ and ‘paranoia’), frequencies were higher in the Nigerian sample compared to the Dutch and Norwegian samples, which were similar in this regard, as hypothesized. However, contrary to expectations, Nigerian participants reported comparable or even higher levels of psychotic experience related distress than did Dutch and Norwegian participants, not fitting the notion of psychotic phenomena as constituting a relatively more normative and hence less distressing aspect of human experience in Nigerian compared to Dutch and Norwegian culture.

Our findings suggest, first of all, that caution may be warranted when comparing frequency and distress of CAPE-*Pos* experiences between general population samples from different countries using available translations of CAPE-42, as observed differences may partly reflect differences in the measure rather than in the latent construct(s). Measurement non-invariance may be due to a variety of differences between samples, e.g. differences in the applicability of items, the conceptual meaning or understanding of constructs, the extent of social desirability, the nature of personal reference points and extreme item responses, item translations, or due to different administration methods [79]. Previous work [64] has suggested that individuals with psychosis vulnerability may show a tendency of scoring slightly lower on CAPE-42 when the measure is administered online versus on paper, but concluded this to be of negligible consequence for research in general population samples. Nonetheless, considering the CAPE-42 questionnaire was assessed online by Dutch and Norwegian participants, but on paper by Nigerian participants, differences in the administration method may have represented a source of measurement non-invariance. Moreover, CAPE-42 was developed in the Netherlands and it cannot be ruled out that Dutch values, beliefs, and attitudes have influenced the idiom of the questions [22], and may have been understood differently by Norwegian and Nigerian participants presented with translated versions of the instrument. Assessment tools may not always be applicable cross-culturally [85], and our findings may imply that further cross-cultural adaptation of CAPE-42 for non-Western samples may thus be necessary [86] (see also ITC Guidelines for Translating and Adapting Tests [87]).

Despite differences in the measurement of CAPE-42 positive psychotic experiences across cultural settings, similarities were observed for the measurement of ‘strange experiences’ and ‘paranoia’, of which the observed higher frequency in Nigerian participants aligns with previous work showing higher psychotic symptom prevalence of non-Western compared to Western societies [13, 22, 33, 42, 53, 54]. As suggested previously [16, 50], a more pliable way of differentiating between reality and fantasy in non-Western societies–expressed, for example, through recognition of a supernatural or divine realm–may give rise to more frequent endorsement of psychotic-like experiences, such as imagery, or altered states of consciousness, than in Euro-American societies, in which distinctions between what is real and what is not tend to be more rigid [50]. Experiences such as communicating with a deceased family member or feeling the presence of a supernatural force may in fact be highly valued and culturally meaningful in non-Western societies [88], and may therefore be more frequently noticed and more easily communicated to others [50]. A higher prevalence of positive psychotic symptoms in African societies may thus, in part, reflect a greater tendency to share culturally sanctioned experiences that signify contact with the supernatural realm or spirit world [38, 50, 60]. In Western societies, on the other hand, negative attitudes towards experiences that could signal dissociation from what is sensible or physically perceivable (i.e., what is considered as ‘real’), may reduce the tendency to report such experiences, out of fear for stigma of mental illness [50].

However, while the Nigerian participants included in our study reported a higher frequency of positive psychotic experiences, they also reported equal or even higher levels of distress due to these experiences compared to Dutch and Norwegian participants, respectively. This does not seem to fit well with the idea of psychotic-like experiences representing a more normative and benign, and hence less distressing aspect of human experience in non-Western societies [16, 20, 22, 50]. Rather, this finding suggests that the experience of psychotic phenomena may be just as, if not more, distressing in African than in European culture. This contrasts with previous reports, describing for instance that hallucinations are considered less troublesome and in general are experienced positively in African culture [20], in addition to suggesting more sympathetic attitudes within the social environment, a lower demand for clinical support, a more transient nature, and favourable course and outcome of psychosis in non-Western societies [85, 89–93], although some studies suggest that symptom severity rather than associated distress is linked to need for care [94]. However, other work suggests that prevalence-distress associations for psychotic symptoms do not necessarily differ across cultural groups [95], and certain culture-specific spiritual or supernatural interpretations of psychotic-like experiences, such as beliefs of possession by higher order malevolent entities (typically: devils, demons) as described for certain social groups in Africa, may actually induce severe distress [96, 97]. Reversely, the experience of psychological distress may be expressed or communicated differently across cultures [98, 99], and the endorsement of psychotic-like symptoms in Africans may in fact reflect a culturally sanctioned expression of distress, in line with the observation of higher rates of brief reactive psychoses following stressful events in non-Western compared to Western cultures [100].

Because the current study did not tap directly into the belief system of participants, it remains unclear exactly in which way and to what extent culturally based beliefs drive the findings on psychotic symptom frequency and distress. Several studies have suggested, for instance, that while on the one hand Nigerians are in general acculturated in the sense that they support attempts to emulate western economic and social developments, they are on the other hand very protective of traditional cultural values and norms [101, 102]. This may explain why caregivers of schizophrenia patients in Nigeria endorse both supernatural (‘traditional’), as well as natural (‘acculturated’) causes as important in the aetiology of the disorder [103], and why Nigerian patients with schizophrenia seek help from both spiritual healers and physicians [104]. Nigerians may thus represent a relatively heterogeneous group in terms of beliefs and attitudes regarding psychotic experiences, with the possibility of contrasting or bicultural belief systems at play. Educational level may play a role, as supernatural beliefs about disease causation are less commonly endorsed by individuals with formal education [103]. This may well be relevant for Nigerian participants in the current study, given that all had completed secondary or tertiary education. Moreover, respondents were psychology students with expectedly higher tendency towards medical and scientific explanations for their experiences, making them more aware of possible psychopathology.

Apart from cultural factors, the participant samples included in the current study may have differed in terms of demographic and exposure to environmental risk factors for psychosis. The Nigerian sample, for instance, consisted of proportionally more men, was significantly younger, lower educated, and less often married than the Dutch and Norwegian samples. Therefore, although statistical analyses were corrected for demographic variability, it cannot be ascertained that between-group differences do not in part reflect different demographic risk profiles for psychosis [8]. In addition, previously identified environmental risk factors for psychosis, such as childhood trauma, cannabis use, and urbanicity may have differed between samples but were not assessed [44]. Whereas lifetime cannabis use is likely more common in Norway, and particularly in The Netherlands, compared to Nigeria (see e.g. [105]), reports of childhood abuse may be more commonly reported by African patients with a psychiatric diagnosis compared to patients from Europe [106, 107]. With regard to urbanicity, it is important to recognize that Nigerian participants were from the city of Lagos, which is considered the fastest growing and largest city of Africa, with an estimated population of over 20 million residents [108]. Moreover, the city has been ranked among the least liveable cities in the world, due to issues regarding political stability, security, healthcare, education, and infrastructure [109]. Urban residency was previously shown to be associated with delusion-like experiences in young Ugandan adults, regardless of age, gender and social class [52]. Thus, although speculative, between-group differences in reported rates of psychotic experiences and associated distress may be partly attributable to differences in exposure to environmental risk rather than differences in cultural background.

Although reported frequencies of positive psychotic experiences were comparable between Dutch and Norwegian participants, our data suggest that experiences of paranoia caused less distress for Norwegian than for Dutch students. Although this finding agrees with previous work showing that psychological distress in general tends to be lower in Norwegian students compared to students from other Western countries [110], our study is the first to compare psychosis proneness-related distress between Dutch and Norwegian adults, and further investigations are required to test the robustness of this result, and elucidate possible involvement of cultural factors.

### Strengths and limitations

This study adds to the existing literature on subclinical psychotic experiences in community-based samples, by assessing measurement invariance and differences in frequency and distress of CAPE-42 positive psychotic experiences across student samples from different cultural settings. In particular, the current study responds to the dearth of CAPE-42 data for non-Western study samples (see [25] for recent overview), by exploring CAPE-42 positive psychotic experiences in African students. Although the current study thereby provides new insights in the extended psychosis phenotype across sociocultural groups, a number of limitations require consideration when interpreting results, and deserve attention in future studies.

First, as pointed out above, the Nigerian study sample differed significantly from the Dutch and Norwegian samples regarding demographic characteristics, and possibly also regarding exposure to environmental (e.g. trauma, cannabis use, urbanicity [44]) and other risk factors for psychosis, such as co-occurring phenomena like anxiety and depression [111, 112]. Therefore, although findings were statistically adjusted for demographic characteristics, caution is warranted when attributing between-group differences to cultural factors. Second, higher educated individuals were overrepresented in all samples, and the Dutch and Norwegian samples consisted largely of women. In addition, recruitment procedures may have led to selective underrepresentation of certain groups in our study sample, and results thus possibly lack accuracy regarding generalization to the population level (see e.g. [113, 114] for potential limitations of non-probability sampling). Third, although we assumed similarities between Dutch and Norwegian participants with respect to their cultural belief system in context of distinctions between reality and fantasy, they cannot be interpreted as culturally equal. Fourth, the current study did not distinguish between self-reported ethnic group membership of Nigerian participants. Fifth, (partial) measurement invariance of CAPE-*Pos* across samples was only established for items pertaining to ‘strange experiences’ and ‘paranoia’, thus limiting between-group comparisons to these dimensions of CAPE-*Pos*. In addition, because reports of experience related distress were selectively available–only for those experiences labelled as present–measurement invariance testing for the distress dimension was omitted, in line with previous work [64]. As discussed above, differences in administration, language, and interpretation of CAPE-42 between samples all represent potential sources of measurement non-invariance in our study, and subject to further investigation in future cross-cultural studies using CAPE-42. Sixth, internal consistency of CAPE-*Pos* subdimensions supported by CFA varied from ω = 0.30 to ω = 0.82, and findings must be interpreted in this context. Seventh, the current study did not include an infrequency scale (e.g. [115]) or other measures for identifying inconsistent survey responders, thus the possibility of dishonesty, survey fatigue, or other response biases affecting our data cannot be ruled out. Eighth and lastly, this study focused on the positive dimension of psychotic experiences, because it was considered most sensitive to cross-cultural differences in belief systems [17, 50]. However, recent work by [116] suggested a multidimensional nature of the extended psychosis phenotype in which positive and negative psychotic experiences, disorganization, mania and depression complement a general transdiagnostic psychosis factor (i.e., a factor that is relevant across a range of mental disorders). Future cross-cultural studies may thus consider a more comprehensive assessment of the extended psychosis phenotype that takes this multidimensionality into account.

## Conclusion

Our findings show cultural variation in the extended psychosis phenotype, and support previous work suggesting that culture may profoundly affect various dimensions of psychometric measures of psychosis proneness [16]. The results emphasize the importance of investigating cross-cultural variants in symptom definition, and behavioural and symptomatic manifestations of psychosis [117]. Awareness of the sociocultural context in which psychotic experiences occur is required in order to adequately interpret these experiences and respond appropriately [12, 14, 15, 118–120]. Prevention, screening, identification, and treatment of psychosis in non-Western cultural settings may require involvement of and collaboration between traditional healers and health professionals, as already operationalized in certain African countries for HIV/AIDS and related illnesses [121–123].

Future studies are advised to further investigate and minimize sources of measurement non-invariance, explicitly examine cultural acceptance towards psychotic phenomena, and assess environmental and other known risk factors for psychosis when comparing and interpreting subclinical psychotic phenomena across different cultural groups.

## Data Availability

The dataset is available from the corresponding author on reasonable request.

## Appendix

**Table 7:** Confirmatory Factor Analysis for the total sample, and for the three study samples separately

CAPE-*Pos* frequency				Absolute goodness of fit			Comparative fit		
	χ^2a,b^	df^c^	*p*	RMSEA^d^ (90% CI)	CFI^d^	SRMR	AIC^b^	BIC^b,e^	Factor loadings
Whole sample (*n* = 786)^f^									
1-factor model^g^	677	152	< 0.001	0.082 (0.075; 0.088)	0.824	0.060*	29,905	29,962	0.307–0.681
3-factor model^h^	459	149	< 0.001	0.063 (0.056; 0.069)*	0.898	0.050*	29,566	29,627	0.316–0.786
5-factor model^i^	320	142	< 0.001	0.048 (0.041; 0.056)*	0.942*	0.041*	29,372	29,443	0.348–0.904
Dutch sample (*n* = 245)^f^									
1-factor model^g^	344	152	< 0.001	0.082 (0.071; 0.094)	0.573	0.085	4035	4048	0.029–0.541
3-factor model^h^	317	149	< 0.001	0.074 (0.063; 0.086)*	0.656	0.083	3973	3987	0.278–0.679
5-factor model^i^	218	142	< 0.001	0.051 (0.037; 0.064)*	0.845	0.068*	3868	3884	0.299–0.988
Nigerian sample (*n* = 380)^f^									
1-factor model^g^	407	152	< 0.001	0.073 (0.064; 0.081)*	0.742	0.070*	16,591	16,620	0.103–0.580
3-factor model^h^	314	149	< 0.001	0.059 (0.049; 0.068)*	0.836	0.063*	16,478	16,510	0.143–0.728
5-factor model^i^	254	142	< 0.001	0.050 (0.040; 0.059)*	0.888	0.053*	16,423	16,460	0.217–0.737
Norwegian sample (*n* = 161)^f^									
1-factor model^g^	359	152	< 0.001	0.100 (0.087; 0.114)	0.455	0.103	3904	3901	0.095–0.533
3-factor model^h^	316	149	< 0.001	0.087 (0.074; 0.100)	0.600	0.094	3825	3822	0.185–0.944
5-factor model^i^	301	142	< 0.001	0.086 (0.072; 0.099)	0.626	0.091	3816	3812	0.211–0.971

^a^Yuan-Bentler scaled test-statistic, ^b^Rounded to nearest integer, ^c^Item no. 41 dropped, due to (near-)zero variance in Dutch and Norwegian samples (i.e. [virtually] all scores = 1), ^d^Robust RMSEA and CFI from the scaled test-statistic, ^e^Sample size adjusted Bayesian BIC, ^f^Missing data points were processed using listwise deletion, ^g^CAPE-*Pos* one-factor structure as originally reported by [67], ^h^CAPE-*Pos* three-factor structure as reported by [25], ^i^CAPE-*Pos* five-factor structure as reported by [26], *Meeting minimally acceptable fit criteria: CFI ≥ 0.90 [75], RMSEA ≤ 0.08 [76], SRMR ≤ 0.08 [77]

**Table 8:** Fit statistics for different invariance models of CAPE-42 positive psychotic experiences across study samples

CAPE-*Pos* frequency	χ^2^ (Δχ^2^)^a^	df (Δdf)	*p* (Δ*p*)	CFI (ΔCFI)^b^	RMSEA (ΔRMSEA)^b^	SRMR (ΔSRMR)	Comparison	Decision
5-dimensional model^c^; *n* = 245^d^ (Netherlands); *n* = 478^d^ (Nigeria); *n* = 162^d^ (Norway).								
M1 Configural invariance	768	426	< 0.001	0.823	0.059	0.063	–	Reject
M2 Metric invariance	809 (41)	454 (28)	< 0.001 (0.018)	0.804 (0.019)	0.061 (−0.002)	0.074 (−0.011)	M1 vs. M2	Reject
M3 Scalar invariance	1004 (195)	482 (28)	< 0.001 (< 0.001)	0.711 (0.093)	0.071 (− 0.010)	0.088 (− 0.014)	M2 vs. M3	Reject
2-dimensions reduced model (Strange experiences – items 5, 17, 24, 26, 28, 30, 31; Paranoia – items 2, 6, 7, 22); *n* = 245^d^ (Netherlands); *n* = 478^d^ (Nigeria); *n* = 162^d^ (Norway).								
M1 Configural invariance	197	129	< 0.001	0.917	0.049	0.050	–	Accept
M2 Metric invariance	213 (16)	147 (18)	< 0.001 (0.291)	0.911 (0.006)	0.048 (0.001)	0.062 (−0.012)	M1 vs. M2	Accept
M3 Scalar invariance	334 (121)	165 (18)	< 0.001 (< 0.001)	0.775 (0.136)	0.071 (−0.023)	0.086 (−0.024)	M2 vs. M3	Reject
M3a Partial scalar invariance^e^	226 (13)	159 (12)	< 0.001 (0.375)	0.910 (0.001)	0.046 (0.002)	0.065 (−0.003)	M2 vs. M3a	Accept

ΔCFI < 0.01 implies that invariance assumption holds [81], ^a^Yuan-Bentler scaled test-statistic (rounded to nearest integer), ^b^Robust RMSEA and CFI from the scaled test-statistic; ^c^Item no. 41 dropped, due to (near-)zero variance in Dutch and Norwegian samples (i.e. [virtually] all scores = 1); ^d^Missing data points were processed using listwise deletion, ^e^No intercept constraints for items 6, 7, 17

## Abbreviations

AIC: Akaike information criterion
BIC: Bayesian information criterion
BPRS: Brief Psychiatric Rating Scale
CAPE-42: Community Assessment of Psychic Experiences
CAPE-*Pos*: CAPE positive symptom dimension
CFA: Confirmatory factor analysis
CFI: Comparative fit index
DSM-(I)V: Diagnostic and Statistical Manual of Mental Disorders IV / V
FIML: Full information maximum likelihood
ICD-10: International Classification of Diseases-10
MANCOVA: Multivariate analysis of covariance
MGCFA: Multi-group confirmatory factor analysis
RMSEA: Root mean square error of approximation
SIS-R: Structured Interview for Schizotypy
SPSS: Statistical Package for the Social Sciences
SRMR: Standardized root mean square residual
